# Metagenomic Analysis Reveals the Distribution of Antibiotic Resistance Genes in a Large-Scale Population of Healthy Individuals and Patients With Varied Diseases

**DOI:** 10.3389/fmolb.2020.590018

**Published:** 2020-10-30

**Authors:** Qinwei Qiu, Jingjing Wang, Yuhong Yan, Bhaskar Roy, Yang Chen, Xiaoxiao Shang, Tongyi Dou, Lijuan Han

**Affiliations:** ^1^State Key Laboratory of Dampness Syndrome of Chinese Medicine, The Second Affiliated Hospital of Guangzhou University of Chinese Medicine, Guangzhou, China; ^2^Guangdong Provincial Hospital of Chinese Medicine, Guangzhou, China; ^3^School of Life and Pharmaceutical Sciences, Dalian University of Technology, Panjin, China; ^4^Department of Scientific Research, KMHD, Shenzhen, China; ^5^BGI Genomics, BGI-Shenzhen, Shenzhen, China

**Keywords:** gut microbiome, antibiotics, antibiotics resistance genes, resistome, metagenomics

## Abstract

The human gut microbiome is a reservoir for antibiotic resistance gene (ARG). Therefore, characterizing resistome distribution and potential disease markers can help manage antibiotics at the clinical level. While much population-level research has highlighted the strong effect of donor geographic origin on ARG prevalence in the human gut, little is known regarding the effects of other properties, such as age, sex, and disease. Here we employed 2,037 fecal metagenomes from 12 countries. By quantifying the known resistance genes for 24 types of antibiotics in each community, we showed that tetracycline, aminoglycoside, beta-lactam, macrolide-lincosamide-streptogramin (MLS), and vancomycin resistance genes were the dominant ARG types in the human gut. We then compared the ARG profiles of 1427 healthy individuals from the 2,037 samples and observed significant differences across countries. This was consistent with expectations that regional antibiotic usage and exposure in medical and food production contexts affect distribution. Although no specific uniform pattern of ARG was observed, a significant increase in resistance potential among multiple disease groups implied that the disease condition may be another source of ARG variance. In particular, the co-occurrence pattern of some enriched bacterial species and ARGs that were obtained in type 2 diabetes (T2D) and liver cirrhosis patients implied that some disease-associated species may be potential hosts of enriched ARGs, which could be potential biomarkers for the prediction and intervention of such diseases. Overall, our study identifies factors associated with the human gut resistome, including substantial effects of region and heterogeneous effects of disease status, and highlights the value of ARG analysis in disease research and clinical applications.

## Introduction

Antibiotic resistance in pathogens is a global health crisis (Klein et al., 2018). Abuse of antibiotics in clinical settings and food production results in the widespread adaptation of bacteria to antibiotic exposure, as well as to the rapid evolution and dissemination of antibiotics resistance genes (ARGs). The presence of ARGs generally indicates lower antibiotic susceptibility in pathogens, making these genetic markers valuable for clinical screening.

A tremendous number of microbes inhabit the human intestinal tract and play a vital role in human physiological function (Lynch and Pedersen, 2016). The human gut microbiome is considered a reservoir of ARGs, which makes it possible to investigate clinical resistance by gut microbiome analysis (Haak et al., 2018). Various approaches, such as the isolation of antibiotic resistant bacterial strains, DNA microarrays (Lu et al., 2015), and metagenomic expression libraries, have been used to characterize the resistome within a community (Boolchandani et al., 2019). Among these strategies, high-throughput sequencing-based metagenomic analysis is a powerful tool for ARG surveys. Several comparative resistome studies have been performed to date using sequence data analysis from fecal samples (Forslund et al., 2013; Hu et al., 2013; Gibson et al., 2015; Li et al., 2015, 2018; Rampelli et al., 2015; Fitzpatrick and Walsh, 2016; Pal et al., 2016; Pehrsson et al., 2016; Feng et al., 2018). Four significant from this research include: (i) The most abundant resistance determinants in the human gut are those for antibiotics available for a long time (Forslund et al., 2013). (ii) Robust differences in country-level resistance potential are observed, which correlate with local antibiotic consumption (Forslund et al., 2013; Hu et al., 2013; Pehrsson et al., 2016). (iii) Donor properties other than country of origin, such as age, sex, or body mass index (BMI), have minor influences on the resistome (Forslund et al., 2014) (iv) Resistance potential is significantly correlated with microbial community composition (Pehrsson et al., 2016; Feng et al., 2018).

However, most of these previous studies suffer from small sample size, which reduces the statistical power of the analyses. In thee integrated analyses of multiple data sets, generalized results demand increased sample sizes to overcome the ambiguous comparisons. Furthermore, the resistome in disease states is largely unexplored. Antibiotic intervention influences some phenotypes closely related to the gut microbiome, such as metabolic disorders (Yang et al., 2017; Fu et al., 2018), immunopathologies (Wypych and Marsland, 2018), cancer (Cao et al., 2018; Zhang et al., 2019), and hypertension. Thus, it is worthwhile to explore the characteristics of ARGs in disease states and its association with bacteria, which may provide a new perspective for the identification of disease markers.

In this study, we aim to explore the factors associated with ARG’s composition in a large samples size. By description of the ARG profiles of 2,037 individuals from 12 countries, we provide an overview of the human gut resistome. This investigation will enhance our understanding of ARG’s population distribution.

## Materials and Methods

### Data Sets

A total of 2,037 fecal metagenome samples from 1,004 Chinese, 32 Japanese, 62 Mongolian, 463 Israelis, 15 Austrian, 55 French, 239 British, 41 Swedish, 24 Canadian, 19 Peruvian, 32 EI Salvadoran, and 51 American (United States) individuals, were collected from the National Center for Biotechnology Information (NCBI) Sequence Read Achieve (SRA^1^) (Leinonen et al., 2011) and our unpublished datasets. Among them, 1,427 samples were from healthy individuals, while 610 Chinese samples were from disease state individuals. Seven disease states were surveyed: colorectal cancer (CRC), type 2 diabetes (T2D), liver cirrhosis, rheumatoid arthritis (RA), (pre)hypertension, psoriasis (our unpublished dataset), and ankylosing spondylitis (AS). All sequencing data were generated using pair-end shotgun sequencing by Illumina technology. Physiological data of all subjects, including age, sex, BMI, and health status were extracted from literature sources and are listed in Supplementary Table S1.

### ARGs and Microbial Taxon Quantification

ARG profiles were acquired with the ARGs-OAP v. 2.0 pipeline (Yin et al., 2018). This pipeline consists of a regularly updated database, Structured Antibiotic Resistance Genes (SARG), that has a hierarchical structure (ARG type-subtype-reference sequence). SARG is an integrated database containing sequences from two other commonly used resistance gene databases, ARDB (Antibiotic Resistance Genes Database) and CARD (The Comprehensive Antibiotic Resistance Database). SARG version 2 is further complemented using a potential resistance protein collection from the NCBI-NR protein database. The abundance of a gene (unit: number of ARG sequences in one million sequences) is the ARG-like sequence number normalized to the corresponding ARG reference sequence length (nucleotide) and the total reads in each metagenomic sample. MetaPhlAn2 (Segata et al., 2012) was used to characterize taxonomic profiles of the sample fecal metagenomes.

### ARG Total Abundance and Diversity Comparison

Both the total abundance and the diversity of resistance genes were considered. Richness of all subtypes was defined as the diversity in a sample. The total ARG abundance was obtained by using the sum of the normalized values of all subtypes. These two indices were compared between populations using Kruskal–Wallis and Wilcoxon sum-rank tests. ARG structure was visualized using non-metric multidimensional scaling (NMDS) analysis based on the abundance matrix of the subtypes, and then tested by permutational analysis of variance (PERMANOVA) from the R vegan package (Oksanen et al., 2019). The R vegan adonis function with 999 permutations and the Bray–Curtis method were used to calculate pairwise distances after the samples with missing phenotypic value were removed.

### Identification of Discriminative ARGs and Enriched Bacteria

Specific indicator subtypes for each population were identified using the indval function from the R Laboratory for Dynamic Synthetic Vegephenonenology (labdsv) package (Roberts, 2019), where IV ranges from 0 to 1 with higher values for stronger indicators. ARG subtypes with IV value > 0.3 and *p*-value < 0.05 in a specific group were selected as potential indicators. Linear discriminant analysis effect size (LEfSe) (Segata et al., 2011) was used to identify enriched bacteria between healthy and disease groups, with the alpha value set for class normality and the threshold of Wilcoxon sum-rank test set to 0.05. The threshold on the logarithmic score of the linear discriminant analysis (LDA) was set to 2.0.

### Association Analysis of Bacteria and ARGs

The Procrustes test for correlation analysis between ARGs and bacterial communities was performed in R with the vegan package (Oksanen et al., 2019). Spearman’s correlation analysis was employed to assess associations between ARG subtypes and bacterial species. Pairs with thresholds of correlation coefficient > 0.5, adjust *p*-value < 0.05, and with the two items both occurring in more than half of the samples were selected to build the network using software Gephi (Bastian et al., 2009).

## Results

### Overview of ARGs Detected in the Human Gut

**FIGURE 1:**
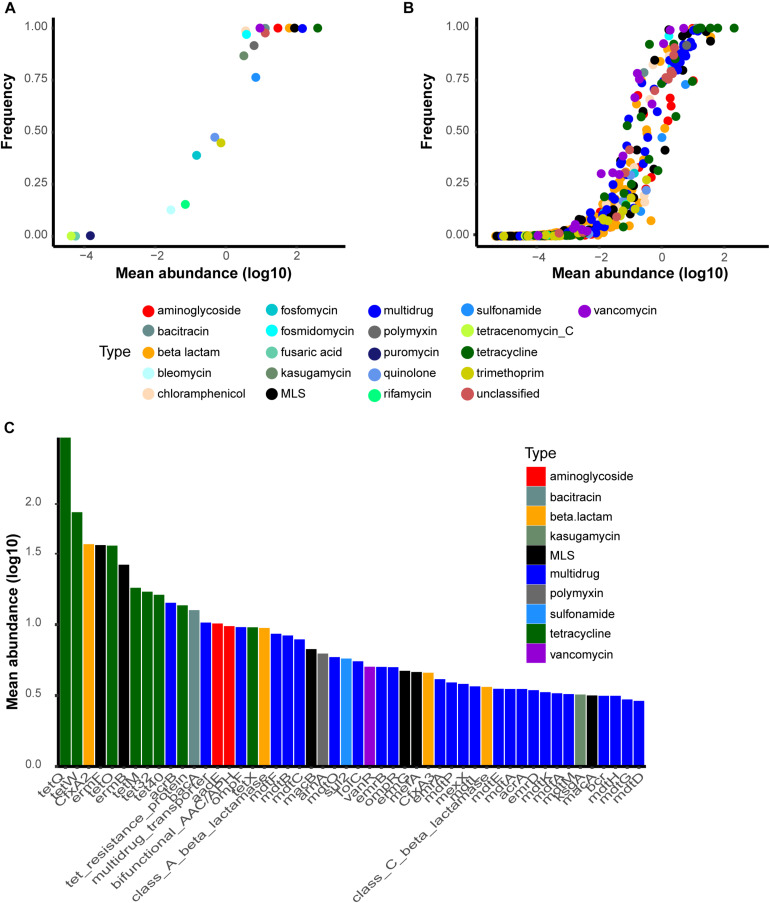
Most common antibiotic resistance gene (ARG) types and subtypes found in the present study. The *y*-axis is the detection rate and the *x*-axis represents the log10-transformed mean abundance of each ARG **(A)** types and **(B)** subtypes in all samples. **(C)** Top50 most abundant subtypes in the human gut. Abbreviations: bifunctional_AAC/APH, bifunctional aminoglycoside N-acetyltransferase and aminoglycoside phosphotransferase.

There are 24 ARG types and 1208 subtypes in SARG v.2. We found 21 of 24 types, in total, in at least one of the 2,037 samples. The human gut resistomes that we analyzed were dominated by particular ARG types with high prevalence and abundance. For example, genes conferring resistance toward tetracycline, aminoglycoside, beta-lactam, macrolide-lincosamide-streptogramin (MLS), and vancomycin were shared by all samples (Figure 1A). Other common resistance gene types included bacitracin, multidrug, chloramphenicol, fosmidomycin, and polymyxin. However, there was an absence of carbomycin, fusidic-acid and spectinomycin resistance genes. ARGs resistant to puromycin, tetracenomycin_C, and fusaric-acid were considered rare types because of low detected rates, 0.30%, 0.15%, and 0.10%, respectively. At the subtype level, 809 subtypes were detected in at least one sample. There were 100 ubiquitous ARG subtypes defined by those present in at least half of our samples (Figure 1B). Most of the ubiquitous subtypes belong to the multidrug resistance type (34), followed by tetracycline (15), beta-lactam (10), MLS (9), aminoglycoside (8), vancomycin (7), and unclassified (7) resistance genes. Six tetracycline resistance genes (*tet32*, *tetM*, *tetO*, *tetQ*, *tetW*, and *tet resistance protein*) were detected in all 2,037 samples, indicating widespread occurrence in the human gut. Additional dominant subtypes that we found in the human microbiome included *bacA* (within the bacitracin type), *vanR* (within the vancomycin type), *aadE* (within the aminoglycoside type), *CfxA2* (within the beta-lactam type), *ermF* and *ermB* (within the MLS type), and *acrB* (within the multidrug) (Figure 1C). The abundance of ARG types and subtypes that we found is shown in Supplementary Tables S2, S3.

### Geographic Origin and Health Status Impacting on ARGs

Geographic factors are widely accepted to be associated with ARGs profile (Forslund et al., 2013; Hu et al., 2013; Pehrsson et al., 2016). To verify this hypothesis, a permutational multivariate analysis of variance (PERMANOVA) was applied based on the subtype profile of 2,037 samples. The result confirmed that geographic origin was a strong influential factor impacting on ARGs (*R*^2^ = 0.2, *p*-value = 0.001). The result also suggested a significant difference across health states with a *p*-value of 0.001, although it was much weaker than geographic origin (*R*^2^ = 0.05). ARG baseline distribution and its alteration in disease groups were described in subsequent sections.

### The Gut ARG Distribution Profile Baseline in Healthy Individuals

**FIGURE 2:**
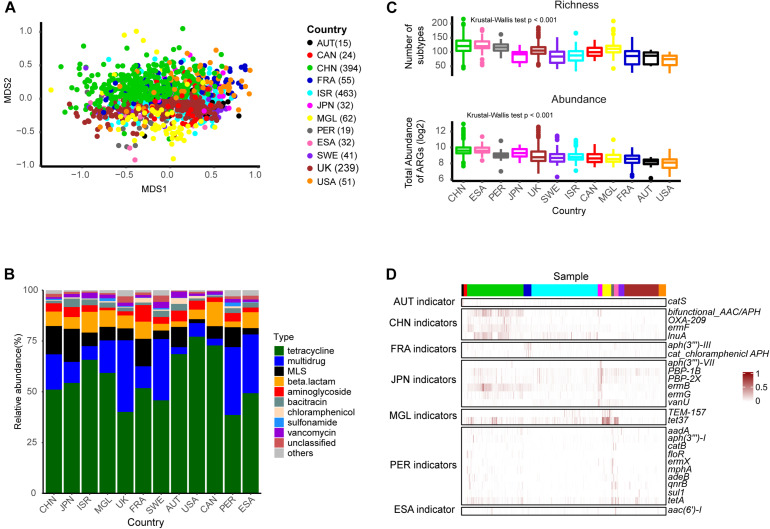
The ARG profile in healthy individuals across different countries. **(A)** Non-metric multidimensional scaling (NMDS) plot based on subtype profiles of 1,427 healthy individuals. **(B)** Composition of types. **(C)** Richness of subtypes and total resistance potential across countries. **(D)** Heatmap of scaled abundance for the 26 indicator subtypes (row) in each sample (column). The number in the brackets indicated the corresponded number of individuals. Abbreviations: AUT, Austria; CHN, China; CAN, Canada; ESA, EI Salvador; FRA, France; ISR, Israeli; JPN, Japan; MGL, Mongolia; PER, Peru; SWE, Sweden.

We visualized subtype profile patterns using NMDS to evaluate resistome composition similarities among 1,427 health samples. The results revealed that resistance subtype profiles were structured by country but with limited overlap (Figure 2A, PERMANOVA analysis: *R*^2^ = 0.21, *p*-value = 0.001), which implied that the distribution of antibiotic resistance genes and the abundance of those genes were geographically distinct. The five widespread types, tetracycline, multidrug, MLS, aminoglycoside, and beta-lactam accounted for >85% of the total antibiotic resistance genes in each of the 12 countries, but the composition of those types varied across the populations (Figure 2B). For example, the tetracycline type was the most abundant and accounted for more than 50% in the majority of countries, except in the United Kingdom, Sweden, Peru, and EI Salvador, where resistance potentials for multidrug were higher. The United States antibiotic resistome had a relative bias toward aminoglycoside and less MLS, which was in contrast to Mongolia.

We then compared ARG richness and potential resistance across different countries, based on the number of subtypes and total abundance for each sample, respectively. As shown in Figure 2C, there were significant differences among these populations. Chinese and EI Salvador samples presented higher resistance potential than the others, which was consistent with previous studies (Pehrsson et al., 2016; Feng et al., 2018; Li et al., 2018). Region-specific subtypes were identified using the R package labdsv. We identified 26 subtypes according to the criteria of IV > 0.3, *p*-value < 0.05 (Figure 2D and Supplementary Table S4) including six aminoglycoside subtypes [*aac(6′)-I, aadA, aph(3″)-I, aph(3^″′^)-III, aph(3′)-VII*, and *bifunctional aminoglycoside N-acetyltransferase and aminoglycoside phosphotransferase*], six MLS subtypes (*ermB, ermF, ermG, ermX, lnuA, mphA*), four beta-lactam subtypes(*OXA-209, PBP-1B, PBP-2X, TEM-157*), four chloramphenicol subtypes (*catB, catS, floR*, and *cat chloramphenicol acetyltransferase*), two tetracycline subtypes (*tet37, tetA*), one multidrug subtype (*adeB*), one quinolone type (*qnrB*), one sulfonamide (*sul1*), and one vancomycin (*vanU*). Ten markers were enriched in the Peruvian population, which involved a limited sample number in the present study. Japanese and Chinese counts were closely behind, accounting for six and four markers, respectively. Previous studies (Hu et al., 2013) have mentioned that *ermF* was China’s representative ARG subtype. Correspondingly, we also found the *ermF* gene subtype (IV = 0.36, *p*-value = 0.001) within the MLS type to be a Chinese indicator. Another subtype, however, *bifunctional aminoglycoside N acetyltransferase and aminoglycoside phosphotransferase* may be more representative of China, because we found a higher indval value (IV = 0.51, *p*-value = 0.001) for it.

In general, our results based on a large baseline number of samples, were consistent with previous findings: that is, the composition of the human-associated resistome was geographic-specific.

### ARG Abundance and Richness in Most Disease State

To describe bias caused by disease, we focused on the 1,004 Chinese samples from seven independent disease state studies: CRC, T2D, liver cirrhosis, RA, (pre)hypertension, psoriasis, and AS. We first examined whether factors such as sample region, age, sex, or BMI could have confounding effects in a healthy subset of the data population (Table 1). Similar to the above results, the geographic effect was obvious. Conversely, the effect of age, sex, or BMI was not significant. To avoid interference of geographic origin, we analyzed each disease dataset separately.

**TABLE 1:** PERMANOVA analysis based on healthy Chinese population.

**Factor**	**df**	**Sums of sqs**	**Mean sqs**	***F* model**	***R*^2^**	***p*-value**
Region	3	5.19	1.73	14.6	0.13	0.001*
Sex	1	0.09	0.09	0.79	2.3e–3	0.586
Age	1	0.1	0.1	0.83	2.4e–3	0.518
BMI	1	0.07	0.07	0.56	1.6e–3	0.822
Residuals	299	35.46	0.12		0.87	
Total	305	40.91			1	

**FIGURE 3:**
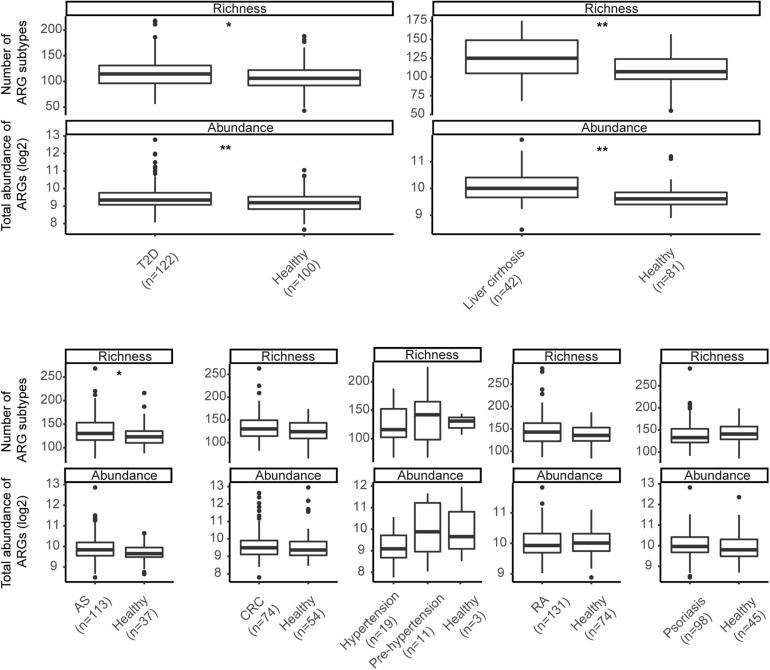
Richness of subtypes and total abundance of ARGs between seven diseases and corresponding controls. Asterisks indicate significant differences (Wilcoxon sum-rank test). * *p*-value ≤ 0.05; ** *p*-value ≤ 0.01.

ARG total abundance and richness were compared in each of the seven datasets, and remarkable differences emerged in most (Figure 3). Both indices in T2D (Richness: *p*-value = 0.019; Abundance: *p*-value = 0.006) and liver cirrhosis (Richness: *p*-value = 0.001; Abundance: *p*-value < 0.001) went beyond those in healthy controls significantly. AS groups had richer ARG diversity (*p*-value = 0.034), while patients with other diseases had no apparent effect on ARG profiles.

### Shared ARG Markers Between Different Diseases

We further identified the ARGs enriched in different disease groups. At the cutoff of IV > 0.3 and *p*-value < 0.05, 147 subtypes were found to be differently enriched between different diseases and corresponding controls (Supplementary Table S5). The T2D and liver cirrhosis group was enhanced by the most subtypes (69 and 67, respectively), while only one subtype (*tetX*) was enriched in the psoriasis group. We could not find any universal pattern of disease markers. Indeed, none of these ARGs were shared by more than three conditions (Supplementary Table S6). The enriched ARGs may reflect the clinical experience of patients. For example, ARGs from the sulfonamide type enhanced in the AS dataset patient group (IV = 0.795, *p*-value = 0.002), consistent with those patients’ exposure to sulfasalazine. A large number of multidrug subtypes was found in patients with liver cirrhosis, many of whom have had a high reported prevalence of multidrug-resistant (MDR) bacterial infection in a previous study (Piano et al., 2019). In general, these results indicated heterogeneous differential ARGs across different disease states.

### Correlation Between Resistance Genes and Microbial Taxa

**FIGURE 4:**
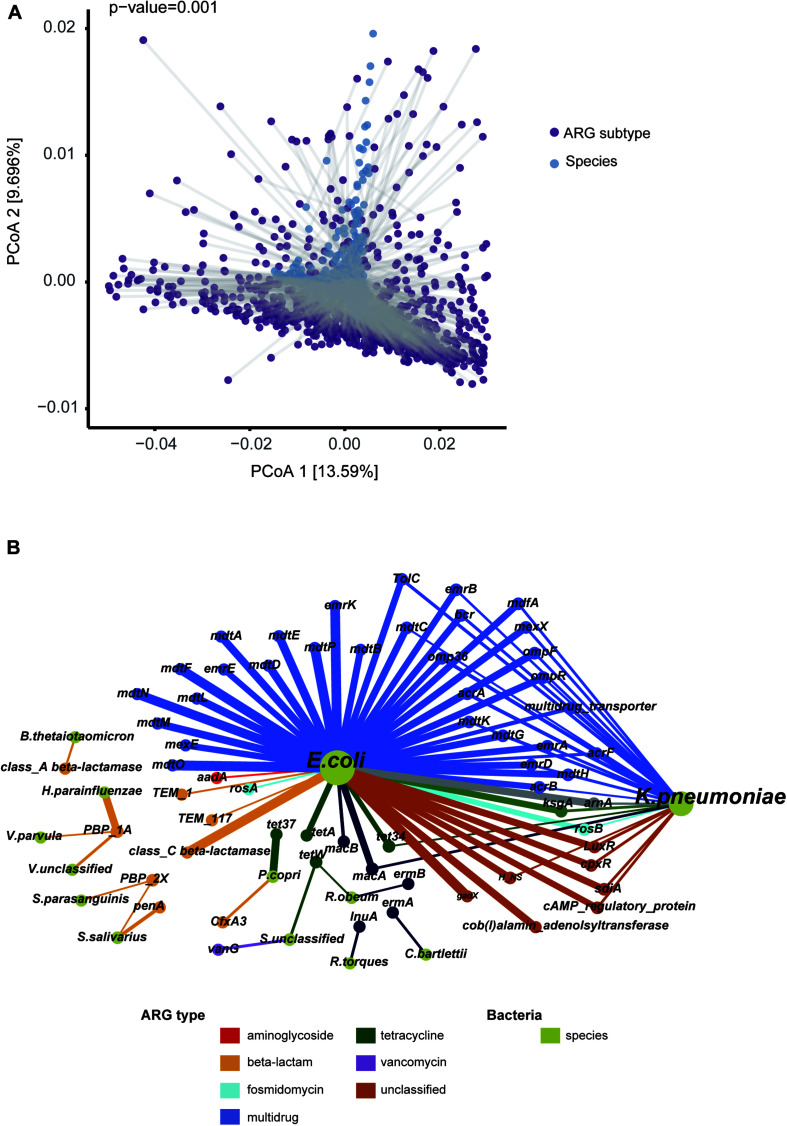
Correlation of ARG subtype and bacteria species. **(A)** Significant agreement between average species and average resistome distances by Procrustes analysis (Monte Carlo *p*-value = 0.001). **(B)** Network representing the co-occurrence patterns between microbial species and ARG subtypes. The chartreuse nodes represent bacterial species. The other nodes represent ARG subtypes, with color according to type. The node size is proportional to degree of occurrence. An edge is a strong (*r* > 0.5) and significant (*p*-value < 0.05) connection between nodes.

Apart from the selective pressure of antibiotics, microbial composition may shape the ARG profile. In support of this notion, our Procrustes analysis showed that give ARG subtypes had significant associations with particular microbial species (Figure 4A, *M*^2^ = 0.912, *p*-value = 0.001). More detailed resistance gene and bacterial phylogeny information was acquired using network analysis. Pairs with Spearman *r* > 0.5 and *p*-value < 0.05 were used to stand for co-occurrence patterns. A total of 74 nodes (13 species and 61 ARG subtypes) and 93 edges (subtypes-species connections) were included in the co-occurrence network (Figure 4B). The resulting figure clearly showed *Escherichia coli* to be a hub node with a high degree of connectedness. It co-occurred with most ARG subtypes, including 31 multidrug resistance genes (e.g., *acrA, acrB, acrF, bcr, emrA, emrB*), seven unclassified type genes (e.g., *LuxR, cpxR, gadX*), and three beta-lactam resistance genes (*class C beta-lactamase*, *TEM_1*, and *TEM_117*).

Relationships between ARG markers and different species, in comparison to that between disease states and controls, were noteworthy. For example, *E. coli* was a T2D-enriched bacteria identified by LEfSe (Supplementary Table S7) and predicted to harbor most of the T2D-enriched subtypes. *Streptococcus salivarius* was more abundant in a liver cirrhosis disease group and might play a key role in promoting cirrhosis with hepatic encephalopathy (Zhang et al., 2013). Our study showed this group to potentially carry beta-lactam resistance subtypes *PBP-2X* (Spearman *r* = 0.502, *p*-value < 0.01) and *penA* (Spearman *r* = 0.607, *p*-value < 0.01). We also found *PBP-2X* to be correlated with another cirrhosis-associated bacteria *Streptococcus_parasanguinis*. Another beta-lactam resistance subtype that we found, *PBP-1A*, was predicted to be carried by *Haemophilus parainfluenzae, Veillonella parvula*, and *Veillonella_unclassified* by our correlation analysis, all of which were enriched bacteria in patients with cirrhosis in our study.

## Discussion

We reanalyzed the metagenomic sequence data of 2,037 samples, focusing on geographic and disease effects on the gut resistome. In general, we found geographic origin to be a substantial factor impacting the composition of human gut resistance genes, which agrees with most previous studies. Disease status effects on the gut resistome were heterogeneous, and further investigations may help unearth the value of ARG in disease research and clinical applications.

As expected, tetracycline, aminoglycoside, beta-lactam, MLS, vancomycin, and multidrug resistance genes were the dominant types in the human gut. Larger sample size would help to identify rare ARGs. Regardless, carbomycin, fusidic-acid, and spectinomycin resistance genes were not detected at all. These three types, coupled with puromycin, tetracenomycin_C, and fusaric-acid, were defined as rare types in our data. The unevenness in ARG abundance and prevalence that we observed may be due to: (i) The way antibiotics are used. For example, dominant antibiotics like tetracycline, aminoglycoside, beta-lactam, and MLS have been widely used in animals-based food productions and in human medical care for a very long time, while fusidic-acid and puromycin are primarily used as scientific tools, not particularly suitable for large-scale application toward medical use and food production. (ii) The limitations of the resistome database itself. Reference sequence counts of rare ARG types in SARGv2 are less than 10. This rare sequence sparsity may be contributed to low detection rates. Regardless, a well-established and up-to-date database needs to be representative of reality, and the number of resistance gene types in the database also needs to be determined by the utilization of antibiotics in the real world. Indeed, SARGv2 contains sequences not only from the two most commonly used resistance gene databases, CARD and ARDB, but also carefully selected and curated sequences from the latest protein collection of the NCBI-NR database. The extensive collection of reference sequences in SARGv2 enables less bias in quantitative and qualitative analyses of ARGs (Yin et al., 2018). Moreover, the knowledge gained from large-scale quantifications of known resistance genes can be used as a proxy for the non-characterized fraction of the resistome in a given environment (Bengtsson-Palme, 2018). Generally, our results reinforce the notion that most ARGs in the human gut are those resistant for widely used antibiotics.

Analysis of ARG distribution in the population provides an important indicator for public health policies. For example, similar patterns of resistance between countries indicate an extensive spread of resistance, and inter-country collaborative mechanisms will be urgently needed for the rational use of antibiotics (Zhang et al., 2006). The widespread ARG subtypes identified in our study, such as *tetQ*, *tetW*, *CfxA2*, *ermF*, *bacA*, and *vanR*, are consistent with previous studies (Seville et al., 2009; Hu et al., 2013; Gibson et al., 2015; Fitzpatrick and Walsh, 2016). We also found some subtypes with region-specific markers that have not been mentioned before, such as the MLS type *lnuA* for China, *catS* for Austria, *aph(3′)-VII* for Japan, and *sul1* for Peru. These findings strengthen the view that geographic origin strongly impacts the composition of the human-associated resistome, and provide some promising indicators for surveillance programs of antibiotic resistance.

In addition to geographic variation, we were concerned about antibiotic resistance in patient groups. Previous studies and our data suggest that bacterial phylogeny could structure the antibiotic resistome (Forsberg et al., 2014). Various diseases are known to be associated with intestinal microbiota alterations, which may be reflected in the resistome. A recent example (Vich Vila et al., 2018) was paradigmatic in this respect. In the large population from 1792 participants consisting of inflammatory bowel disease (IBD), irritable bowel syndrome (IBS) and controls, changes in the microbiome composition in patients had an impact on the antibiotic resistance load, although the majority of individuals were not taking antibiotics. In our current study, total resistance abundance or richness alteration was found in most diseases, but no consistent pattern of ARGs was observed. In liver cirrhosis and T2D, some resistance gene perturbations identified were related to the disease-associated bacteria, while a similar outcome was not seen in CRC or autoimmune disorders. This heterogeneity may be attributed to disease characteristics, various treatment regimens, and/or lifestyles among groups of people. For example, a high prevalence of MDR bacterial infection is common in patients with cirrhosis (Piano et al., 2019), which is in quite good agreement with our finding of the enrichment of many multidrug resistance genes in these individuals. Furthermore, most of AS patients in our dataset experienced treatment with sulfasalazine at some point. Although the washout period between intervention and sampling was at least three months, enrichment of sulfonamides type ARGs is still observed in this group of patients. These phenomena imply that ARGs in disease can provide the following indications: (i) The host bacteria of some resistance genes are associated with the etiology of some non-infectious disease. Antibiotic exposure may make it predominant, potentially leading to disease status. In this context, ARGs may be used as indicators for prediction and antibiotic intervention in these diseases; (ii) Disease-associated microbes, identified merely based on the analysis of differences between groups, may be false positives contributed from antibiotic exposure. Patient groups are more likely to be exposed to medical environments with a variety of drugs, and a long-term effect is pronounced in their gut microbiome. This could partly explain why bacteria that are abundant in some diseases are enriched in the healthy group in other studies. In our research, *Streptococcus parasanguinis* and *Streptococcus salivarius* were associated with cirrhosis but enriched in healthy control in AS dataset. Given these bacteria’s strong association with *PBP-2X* and *penA*, further investigations are needed to validate whether they are involved in the pathological process of cirrhosis.

We acknowledge there are some limitations in the current study. First, the samples come from 12 countries, which might not be representative of all world enough, although the geographic scope of these countries covers Europe, America and Asia. Incorporating the data from more different regions, such as traditional populations in Africa, may help to gain additional insights. Second, the geographic sample distribution is non-homogeneous. In baseline of gut ARG distribution profile in healthy individuals, this may lead to biased identification of region-specific ARG markers, especially those in regions with relatively small sample size. When focusing on the unhealthy population, there are 7 disease groups and all samples are from China, which makes the findings insufficient to generalize. Further investigation with more types of diseases, together with comparison their ARG markers between different cohorts are warranted to reach a stronger and fairer conclusion. Third, we used the Spearman’s analysis to relate ARG subtype and species. The significant positive coefficient may further indicate the host-gene relationship. However, resistant genes can be transferred from one to another by mobile genetic elements (MGEs) in the real world. As a result, some genes cannot be identified merely through correlation analysis, especially those that have been widely distributed in most bacteria. More reliable information of ARG host can be obtained by the genome sequence analysis at strain level. Finally, it would be interesting to see if the degree of population resistome variation is at the same level as the other microbiome function. We will focus on the relationship between ARGs and other gene families in the future work. Nevertheless, our work provides a broad view of the human gut resistome and highlights the value of ARG analysis in disease research and clinical applications.

## Data Availability Statement

The datasets presented in this study can be found in online repositories. The names of the repository/repositories and accession number(s) can be found in the article/ Supplementary Material.

## Ethics Statement

Ethical review and approval was not required for the study on human participants in accordance with the local legislation and institutional requirements. Written informed consent from the participants’ legal guardian/next of kin was not required to participate in this study in accordance with the national legislation and the institutional requirements.

## Author Contributions

QQ designed the project, performed the bioinformatic analyses, drafted the data curation, interpreted the results, and revised the manuscript. JW performed the bioinformatic analyses and drafted the manuscript. BR drafted and revised the manuscript. YY drafted the data curation and interpreted the results. YC and XS performed the bioinformatic analyses and drafted the data curation. LH and TD designed the project and supervised the entire investigation. All authors contributed to the article and approved the submitted version.

## Conflict of Interest

The authors declare that the research was conducted in the absence of any commercial or financial relationships that could be construed as a potential conflict of interest.

## Footnotes

1 http://www.ncbi.nlm.nih.gov/sra

## Supplementary Material

Supplementary Table 1 Sample information.

Supplementary Table 2 ARG type abundance in all samples.

Supplementary Table 3 ARG subtype abundance in all samples.

Supplementary Table 4 The 26 subtype markers of country identified by the R package Laboratory for Dynamic Synthetic Vegephenonenology (labdsv). Those IV value > 0.3 with corresponding *p*-values < 0.05 are listed here.

Supplementary Table 5 The 147 different subtypes between diseases and corresponding controls.

Supplementary Table 6 Resistance genes in disease relative to controls. Solid color is indicative of significant enrichment in the disease group (color) or control group (gray).

Supplementary Table 7 Pairs used to build the network. Bacteria is annotated if it is identified as a different taxa by Linear discriminant analysis effect size (LEfSe).

## Abbreviations

ARG: antibiotic resistance gene
AS: ankylosing spondylitis
BMI: body mass index
CRC: colorectal cancer
IBD: inflammatory bowel disease
IBS: irritable bowel syndrome
MLS: macrolide-lincosamide-streptogramin
PERMANOVA: permutational multivariate analysis of variance
RA: rheumatoid arthritis
SARG: structured antibiotic resistance genes database
T2D: type 2 diabetes.
